# Knowledge, Attitude, and Practice Regarding Fibromyalgia Among Primary Care Physicians in Tabuk, Saudi Arabia

**DOI:** 10.7759/cureus.35097

**Published:** 2023-02-17

**Authors:** Amirah Alatawi, Hassan A Moria, Abdulrahman Arshed Alharfy, Mohammed Jameel Sehly, Jalawi Talal A Alotaibi, Yousef Salem Alshammari, Abdulrahim Oudah A Albalawi, Saif Marzoug Alanazi, Abdulrahman Jameel Sehly

**Affiliations:** 1 Department of Family and Community Medicine, Faculty of Medicine, University of Tabuk, Tabuk, SAU; 2 Department of Surgery, Faculty of Medicine, University of Tabuk, Tabuk, SAU; 3 Department of Internal Medicine, Faculty of Medicine, University of Tabuk, Tabuk, SAU; 4 Faculty of Medicine, University of Tabuk, Tabuk, SAU

**Keywords:** saudi arabia, tabuk, primary health care providers, family physicians, practice, attitude, knowledge, fibromyalgia

## Abstract

Background: Adequately informed family physicians have the greatest potential to correctly identify the diagnosis of fibromyalgia (FM) and develop an initial treatment plan. Therefore, it is substantial to determine the levels of weakness and inaccuracy among primary care physicians regarding FM diagnostic criteria and management strategies.

Aim: This study aimed to assess the knowledge, attitude, and practices regarding FM among primary care physicians in Tabuk, Saudi Arabia.

Methods: This cross-sectional study included family physicians who were board-certified or registered in the family medicine training program and working at the government family healthcare centers in Tabuk. A pre-designed, structured questionnaire was distributed either in written form or as an online survey.

Results: This study included 52 primary healthcare physicians. Twenty-two (42.3%) participants incorrectly recorded localized pain as a diagnostic symptom, and 45 (86.5%) incorrectly recorded nonsteroidal anti-inflammatory drugs (NSAIDs), prednisolone, and/or opioids as drugs that are used for treating FM. Only 59.6% were confident in recognizing the symptoms of FM, and 55.8% were confident in differentiating FM from other similar diseases.

Conclusions: The primary healthcare physicians working in the government’s primary healthcare centers in Tabuk City, Saudi Arabia, have low levels of knowledge about diagnostic criteria and treatment strategies for FM. These findings highlight the need for continuous professional development involving family physicians in the primary healthcare setting with suitable continuous medical education (CME) programs concerning FM.

## Introduction

Fibromyalgia (FM) is a chronic, complex, heterogenous disorder characterized by widespread pain, difficulty sleeping, physical exhaustion, and cognitive problems. The disease has an impact on the affected persons, their families, and society [1]. Fibromyalgia is a relatively common disorder that occurs in all populations all over the world, with a prevalence ranging from 2% to 9% in various countries [2,3].

The American College of Rheumatology (ACR) published the first version of the most commonly used diagnostic criteria for FM in 1990. These original ACR criteria emphasized the necessity of chronic and widespread pain and the presence of tender points that involve tenderness in response to palpation of 11 or more sites out of 18 specified areas [4]. After that, the 2010 update of the ACR diagnostic criteria was published. The updated criteria are simple and practical and do not require the tender point for the diagnosis of FM [5].

The clinical diagnosis of FM can be established by the primary care physicians after complete history taking and physical examination as there is no currently available specific diagnostic laboratory test or biomarker [6]. A survey involving six European countries, Mexico, and South Korea revealed that the diagnosis of FM remains a challenge for physicians, especially general practitioners and psychiatrists [7].

Management of FM includes both pharmacologic and non-pharmacologic interventions. Drug therapy is not mandatory, and health education, aerobic exercise, and cognitive-behavioral therapy are considered the main lines of treatment [8,9].

Studies assessing the awareness, perceptions, and attitudes related to the evidence-based diagnosis and management of FM have revealed large variations among healthcare providers, including rheumatologists, general practitioners, and medical students, not only between different specialties but also within the same specialty [10].

Adequately informed family physicians have the greatest potential to correctly identify the diagnosis of FM and develop an initial treatment plan. Therefore, it is substantial to determine the levels of weakness and inaccuracy among primary care physicians regarding FM diagnostic criteria and management strategies.

This study aimed to investigate the knowledge, attitude, and practices of primary care physicians about FM in Tabuk, Saudi Arabia.

## Materials and methods

Study design, setting, and date

This cross-sectional survey study was conducted at the government’s primary healthcare centers in Tabuk, Saudi Arabia, between October and November 2022.

Sample size and sampling technique

The sample size was calculated using an online calculator; the total number is 59, and the population proportion is 50%. Thus, the sample size was 52 participants with a margin error of 4.72 (https://www.calculator.net/sample-size-calculator.html?type=1&cl=95&ci=5&pp=50&ps=59&x=56&y=16).

The unit of analysis is the family medicine physician, and sampling followed the rule of dual frame sampling for a stand-alone facility survey approach [11]. Therefore, the sampling of health centers followed the procedure of a simple random sampling technique. The updated list of health facilities was used to create a sampling frame to balance the coverage through the allocation of health facilities with the use of an updated map from the Ministry of Health. All 25 government primary healthcare centers in Tabuk City were covered.

Eligibility criteria

Family physicians who were board-certified or registered in the family medicine training program and working at the government family healthcare centers in Tabuk were included. Family physicians who were not board-certified or registered in the family medicine training program and those working at private family healthcare centers were excluded.

Data collection tool

The questionnaire was validated from previously published articles with some minor modifications [12,13]. It comprised 20 questions: four demographic questions includes: age, gender, position, and experience as well as 16 questions that assessed the level of knowledge, sources of education, and knowledge about the ACR diagnostic criteria and treatment of FM. The questionnaires were distributed either in written form or as an online survey. Participants were met in their workplace by the co-authors, and all the family physicians, including residents, registrars, and consultants, were invited to participate after an appropriate explanation of the aim of the study.

Statistical analysis

The data analyses were performed using Statistical Package for Social Sciences (IBM SPSS Statistics), version 26 for Windows (IBM Corp., Armonk, NY, USA). Categorical variables were summarized as frequencies and percentages, and the associations between variables were tested using Pearson's chi-square (X2) for independence or Fisher exact tests as appropriate. A P-value of <0.05 was considered statistically significant.

## Results

The study enrolled 52 primary healthcare physicians. Females constituted 51.9%. The most frequent age group was 28-35 years, which constituted 38.5%. Saudi physicians were 48.1%, while non-Saudis were 51.9%. Residency training R1 (25%) and R2 (21.2%) physicians as well as registrars (21.2%) were the most frequent. Most participants have a clinical experience of two to five years (30.8%) (Table 1).

**Table 1:** Demographic and professional characteristics of the study participants

		N	%
Gender	Female	27	51.9
	Male	25	48.1
Age groups (years)	<28	8	15.4
	28-35	20	38.5
	36-45	17	32.7
	46-55	2	3.8
	>55	5	9.6
Nationality	Saudi	25	48.1
	Non-Saudi	27	51.9
Position	Residency training (R1)	13	25.0
	Residency training (R2)	11	21.2
	Residency training (R3)	4	7.7
	Registrar	11	21.2
	Senior registrar	7	13.5
	Consultant	6	11.5
Clinical experience (years)	<2	8	15.4
	2-5	16	30.8
	6-10	11	21.2
	11-15	9	17.3
	>15	8	15.4

Table 2 shows the knowledge of the study participants about the diagnosis of FM. Three-fourths (75.0%) knew the ACR criteria for the diagnosis of FM, and of those, 71.8% used these criteria to make a diagnosis. Almost all participants who did not use the ACR criteria attributed it to either being hard to master (53.5%) or not being reliable (38.5%). About 79.0% of the participants knew that 11 or more tender points are required by the ACR criteria for the diagnosis of FM. The most frequently recognized correct symptoms of FM were fatigue (75.0%), anxiety and depression (69.2%), widespread pain (63.5%), sleep disturbance (55.8%), headache (46.2%), morning stiffness (40.4%), and cognitive problems (26.9%), whereas 22 (42.3%) participants incorrectly recorded localized pain as a diagnostic symptom.

**Table 2:** Knowledge of the study participants about diagnosis of fibromyalgia

		N	%
Do you know ACR criteria for diagnosis of fibromyalgia?	Yes	39	75.0
	No	13	25.0
If you answered “yes” to the above, did you use it to make a diagnosis?	Yes	28	71.8
	No	11	28.2
If you answered “no” to the above, why don’t you use it?	Hard to master	7	53.8
	Not reliable	5	38.5
	Complicated and hard to handle	1	7.7
How many tender points were required by the ACR?	6	11	21.2
	11	29	55.8
	16	9	17.3
	19	3	5.8
Knowledge about symptoms of fibromyalgia			
Widespread pain		33	63.5
Localized pain		22	42.3
Morning stiffness		21	40.4
Headache		24	46.2
Sleep disturbance		29	55.8
Cognitive		14	26.9
Fatigue		39	75.0
Anxiety and depression		36	69.2
Type of knowledge about symptoms of fibromyalgia			
Correct answers		30	57.7
Incorrect answers		22	42.3

Table 3 demonstrates the knowledge of the study participants about the treatment of FM. Most participants (86.5%) correctly realized that the treatment of FM should be both pharmaceutical and non-pharmaceutical. Concerning pharmaceutical treatment, nonsteroidal anti-inflammatory drugs (NSAIDs) were the most commonly recorded (84.6%), either alone or in combination with other drugs. This was followed by muscle relaxants (53.8%) and selective serotonin reuptake inhibitors (36.5%). Furthermore, 45 (86.5%) incorrectly recorded NSAIDs, prednisolone, and/or opioids as drugs that are used for treating FM. Among the study participants, 32 (61.5%) knew that a combination of two or more non-pharmaceutical modalities should be recommended.

**Table 3:** Knowledge of the study participants about the treatment of fibromyalgia

		N	%
Which of the following treatment strategies do you practice in your clinic?	Both pharmaceutical and non-pharmaceutical	45	86.5%
	Pharmaceutical	5	9.6%
	Non-pharmaceutical	2	3.8%
Knowledge about pharmaceutical treatment			
Nonsteroidal anti-inflammatory drugs		44	84.6%
Muscle relaxants		28	53.8%
Selective serotonin reuptake inhibitors		19	36.5%
Tricyclic antidepressants		14	26.9%
Prednisolone		9	17.3%
Opioids		4	7.7%
Duloxetine antidepressant		10	19.2%
Gabapentin		6	11.5%
Type of knowledge about pharmaceutical treatment			
Correct answers		7	13.5%
Incorrect answers		45	86.5%
Knowledge about non-pharmaceutical treatment			
Physiotherapy		10	19.2%
Cognitive behavioral therapy		8	15.4%
Hydrotherapy		1	1.9%
Aerobic exercise		1	1.9%
Combination of two modalities or more		32	61.5%

The registrars followed by the residency physicians showed higher percentages of correct knowledge about symptoms of FM, treatment strategies, and pharmaceutical treatment compared to the consultants, but there were no significant associations between the level of knowledge and the position of the physicians (all p > 0.05) (Table 4).

**Table 4:** Associations between the position of the primary healthcare physician and the type of knowledge about diagnosis and treatment of fibromyalgia

		Position of the physician												Fisher’s exact test
		R1		R2		R3		Registrar		Senior registrar		Consultant		
		N	%	N	%	N	%	N	%	N	%	N	%	P-Value
Type of knowledge about symptoms	Correct	9	69.2	6	54.5	2	50.0	9	81.8	2	28.6	2	33.3	0.195
	Incorrect	4	30.8	5	45.5	2	50.0	2	18.2	5	71.4	4	66.7	
Type of knowledge about treatment strategies	Correct	10	76.9	11	100.0	4	100.0	8	72.7	6	85.7	6	100.0	0.365
	Incorrect	3	23.1	0	0.0	0	0.0	3	27.3	1	14.3	0	0.0	
Type of knowledge about pharmaceutical treatment	Correct	1	7.7	2	18.2	1	25.0	2	18.2	0	0.0	2	33.3	0.525
	Incorrect	12	92.3	9	81.8	3	75.0	9	81.8	7	100.0	4	66.7	

Table 5 shows no significant association between the types of correct and incorrect knowledge about the symptoms, treatment strategies, and pharmaceutical treatments and the duration of clinical experience (all p > 0.05).

**Table 5:** Associations between the duration of clinical experience of the primary healthcare physician and the type of knowledge about diagnosis and treatment of fibromyalgia

		Clinical experience in years										P-value
		<2		2-5		6-10		11-15		>15		
		N	%	N	%	N	%	N	%	N	%	
Type of knowledge about symptoms	Correct	7	87.5	8	50.0	7	63.6	4	44.4	4	50.0	0.391
	Incorrect	1	12.5	8	50.0	4	36.4	5	55.6	4	50.0	
Type of knowledge about treatment strategies	Correct	6	75.0	12	75.0	11	100.0	8	88.9	8	100.0	0.233
	Incorrect	2	25.0	4	25.0	0	0.0	1	11.1	0	0.0	
Type of knowledge about pharmaceutical treatment	Correct	0	0.0	3	18.8	2	18.2	1	11.1	2	25.0	0.774
	Incorrect	8	100.0	13	81.3	9	81.8	8	88.9	6	75.0	

Table 6 demonstrates that senior registrars (85.7%), registrars (81.8%), and consultants (83.3%) showed significantly higher confidence in recognizing the symptoms of FM than the residents R1 (38.5%), R2 (27.3%), and R3 (75.0%) (p = 0.022). Furthermore, the registrars and the senior residents (R3) were significantly more confident in differentiating FM from other similar diseases than the R1, R2, and consultants (p = 0.042). Rheumatologists were significantly recommended by different positions to deal with FM (p = 0.020).

**Table 6:** Attitude regarding the diagnosis of fibromyalgia according to the position of the physician

		Position of the physician												P-Value
		R1		R2		R3		Registrar		Senior Registrar		Consultant		
		N	%	N	%	N	%	N	%	N	%	N	%	
Do you accept fibromyalgia as a separate and distinct clinical identity?	No	1	7.7	3	27.3	0	0.0	0	0.0	1	14.3	0	0.0	0.353
	Yes	12	92.3	8	72.7	4	100.0	11	100.0	6	85.7	6	100.0	
Do you think that fibromyalgia is primarily a psychological illness?	No	9	69.2	6	54.5	1	25.0	4	36.4	2	28.6	2	33.3	0.403
	Yes	4	30.8	5	45.5	3	75.0	7	63.6	5	71.4	4	66.7	
In your opinion, fibromyalgia is more prevalent in which gender?	Female	12	92.3	9	81.8	3	75.0	11	100.0	6	85.7	6	100.0	0.475
	Male	1	7.7	2	18.2	1	25.0	0	0.0	1	14.3	0	0.0	
Are you confident in recognizing the symptoms of fibromyalgia?	No	8	61.5	8	72.7	1	25.0	2	18.2	1	14.3	1	16.7	0.022*
	Yes	5	38.5	3	27.3	3	75.0	9	81.8	6	85.7	5	83.3	
Are you confident in differentiating fibromyalgia from other similar diseases?	No	8	61.5	8	72.7	1	25.0	2	18.2	1	14.3	3	50.0	0.042*
	Yes	5	38.5	3	27.3	3	75.0	9	81.8	6	85.7	3	50.0	
What is the reason for the low diagnostic rate of fibromyalgia?	A	3	23.1	3	27.3	0	0.0	2	18.2	0	0.0	1	16.7	0.372
	B	8	61.5	8	72.7	3	75.0	7	63.6	4	57.1	2	33.3	
	C	2	15.4	0	0.0	1	25.0	2	18.2	3	42.9	3	50.0	
Where have you learned most about fibromyalgia?	CME programs	2	15.4	1	9.1	0	0.0	1	9.1	0	0.0	0	0.0	0.692
	Post-graduation training	6	46.2	6	54.5	3	75.0	7	63.6	6	85.7	6	100.0	
	Undergraduate knowledge	5	38.5	4	36.4	1	25.0	3	27.3	1	14.3	0	0.0	
In your opinion, which healthcare professional deals with fibromyalgia?	Neurologist	0	0.0	0	0.0	1	25.0	0	0.0	2	28.6	0	0.0	0.020*
	Pain management specialist	4	30.8	1	9.1	0	0.0	1	9.1	4	57.1	0	0.0	
	Psychiatrist	1	7.7	4	36.4	0	0.0	2	18.2	0	0.0	2	33.3	
	Rheumatologist	8	61.5	6	54.5	3	75.0	8	72.7	1	14.3	4	66.7	

Table 7 shows that physicians with clinical experience of less than two years were significantly less confident in recognizing the symptoms of FM (p = 0.045). Moreover, a clinical experience of over 15 years was significantly associated with higher confidence in differentiating FM from other similar diseases (p < 0.001). Most of the residents depended on undergraduate knowledge followed by postgraduate training as a learning source, while registrars and consultants learned mainly from postgraduate training and then continuous medical education (CME) programs, with a significant difference (p = 0.033).

**Table 7:** Attitude regarding the diagnosis of fibromyalgia according to the clinical experience of the physician

		Clinical experience in years										P-value
		<2		2-5		6-10		11-15		>15		
		N	%	N	%	N	%	N	%	N	%	
Do you accept fibromyalgia as a separate and distinct clinical identity?	No	1	12.5	1	6.3	1	9.1	1	11.1	1	12.5	0.983
	Yes	7	87.5	15	93.8	10	90.9	8	88.9	7	87.5	
Do you think that fibromyalgia is primarily a psychological illness?	No	5	62.5	8	50.0	6	54.5	3	33.3	2	25.0	0.548
	Yes	3	37.5	8	50.0	5	45.5	6	66.7	6	75.0	
In your opinion, fibromyalgia is more prevalent in which gender?	Female	7	87.5	13	81.3	11	100.0	9	100.0	7	87.5	0.440
	Male	1	12.5	3	18.8	0	0.0	0	0.0	1	12.5	
Are you confident in recognizing the symptoms of fibromyalgia?	No	7	87.5	6	37.5	2	18.2	3	33.3	3	37.5	0.045*
	Yes	1	12.5	10	62.5	9	81.8	6	66.7	5	62.5	
Are you confident in differentiating fibromyalgia from other similar diseases?	No	8	100.0	6	37.5	6	54.5	3	33.3	0	0.0	<0.001*
	Yes	0	0.0	10	62.5	5	45.5	6	66.7	8	100.0	
What is the reason for the low diagnostic rate of fibromyalgia?	A	1	12.5	4	25.0	2	18.2	1	11.1	1	12.5	0.952
	B	6	75.0	10	62.5	6	54.5	5	55.6	5	62.5	
	C	1	12.5	2	12.5	3	27.3	3	33.3	2	25.0	
Where have you learned most about fibromyalgia?	CME programs	0	0.0	2	12.5	0	0.0	0	0.0	2	25.0	0.033*
	Post-graduation training	2	25.0	12	75.0	9	81.8	7	77.8	4	50.0	
	Undergraduate knowledge	6	75.0	2	12.5	2	18.2	2	22.2	2	25.0	
In your opinion, which healthcare professional deals with fibromyalgia?	Neurologist	0	0.0	0	0.0	1	9.1	1	11.1	1	12.5	0.381
	Pain management specialist	1	12.5	5	31.3	2	18.2	1	11.1	1	12.5	
	Psychiatrist	2	25.0	0	0.0	2	18.2	2	22.2	3	37.5	
	Rheumatologist	5	62.5	11	68.8	6	54.5	5	55.6	3	37.5	

## Discussion

This study aimed to explore the level of awareness, attitude, and practices regarding the diagnosis and management of FM among primary healthcare physicians working in the government’s primary healthcare centres in Tabuk, Saudi Arabia.

This study highlights several weaknesses in the current knowledge of the studied primary health care physicians. There was a lack of awareness about the diagnostic symptoms of FM and a substantial deficit regarding the diagnostic pain feature in FM patients. The study participants also showed insufficient confidence in their abilities to diagnose patients with FM and differentiate it from other similar diseases. Moreover, there were misconceptions about the effective drugs used for managing the symptoms of FM and the non-pharmacologic management strategies. The observed findings reflect defects in the training of physicians on diagnostic criteria and differential diagnoses and the best treatment strategies.

In the present study, knowledge about the symptoms of FM was not sufficiently high. Thus, the reporting of fatigue and anxiety as diagnostic symptoms of FM was 75.0% and 69.2%, respectively. Moreover, we recorded a substantial lack of knowledge regarding the diagnostic pain feature, where 22 (42.3%) participants incorrectly recorded localized pain as a diagnostic symptom.

Knowledge and attitudes about FM among medical practitioners, including physicians, nurses, and technologists/technicians in different regions of Saudi Arabia, have been investigated, and the findings indicate poor knowledge about the diagnosis and treatment of FM [14]. Another study from Saudi Arabia showed little awareness of or confidence in the assessment and management of patients with FM among physical therapists [10]. In Egypt, Zeid and Ibrahim [15] have also recently shown a low level of knowledge about the clinical presentation and treatment modalities of FM among Egyptian family physicians working in primary health care centers. Other studies generally reported that awareness about FM is poor among healthcare providers [16,17].

There were no significant associations between the level of correct knowledge about symptoms of FM, treatment strategies, and pharmaceutical treatment and the position of the physician or the duration of his clinical experience. However, we observed that registrars, followed by the residency physicians, showed higher percentages of correct knowledge than the consultants. A corresponding study found that consultants were aware of FM syndrome, but most of them were skeptical and confused about the diagnostic criteria [18].

The majority of participants (86.5%) correctly realized that the treatment of FM should be both pharmaceutical and non-pharmaceutical. However, there were misconceptions about effective drugs for managing the symptoms of FM. Most (86.5%) respondents incorrectly recorded NSAIDs, prednisolone, and/or opioids as drugs that are used for treating FM. Furthermore, the knowledge about how to tailor non-pharmaceutical treatment was not adequate. Among the study participants, 32 (61.5%) knew that a combination of two or more non-pharmaceutical modalities should be recommended.

It is agreed that the management of FM requires a multidisciplinary approach, involving non-pharmacologic treatments as the primary approach, mainly education, exercise and psychotherapy, and thereafter the pharmacologic treatments [19].

Based on the results from clinical trials, the recommended pharmacologic treatments include pregabalin, duloxetine, milnacipran, amitriptyline, cyclobenzaprine, and, in certain patients, tramadol as therapeutic options under restricted conditions [20].

Regarding the attitude and practices of the study participants, confidence in recognizing the symptoms of FM and differentiating it from other similar diseases was lacking (59.6% and 55.8%, respectively). Furthermore, the physician’s confidence level was significantly lower in residents and those with a clinical experience of fewer than two years than in registrars and consultants. The respondents believed that the reason for the low diagnostic rate of FM is mainly the doctors’ lack of knowledge about FM, the patients' failure to communicate their symptoms, or the common misdiagnosis of other diseases. Similarly, a study from China reported that rheumatologists recognized some of the FM-related symptoms but had limited knowledge of the diagnostic criteria. Eighty percent of the respondents declared they had difficulties treating FM patients [12].

It has been evident that the subjective character of symptoms, the absence of a diagnostic test, and the modest response to treatments remain challenges for treating healthcare professionals in standard clinical practice [9]. As well, the presence of several somatic complaints confounds the condition, and the diagnosis of FM is frequently overlooked [21]. Definitely, a well-informed physician and good communication with the patients could reliably reach valid FM diagnoses [22].

Exploring the opinion of the studied family physicians about the nature and cause of FM and the healthcare professionals who should deal with it revealed that a great percentage (90.4%) accepted FM as a separate and distinct clinical identity, and a similar percentage correctly knew that FM is more prevalent in females. However, more than half (53.8%) incorrectly thought that FM is primarily a psychological illness. In this regard, Amber et al. reported that some practitioners doubted the existence of FM as a specific medical condition and others believed it was linked primarily to psychological origins [23]. Additionally, it has been reported that FM is an idiopathic chronic syndrome and its etiology remains unknown, though it can be precipitated by stressful events or physical/emotional traumas [24].

According to the ACR, FM is considered a rheumatological disease, and rheumatologists and pain physicians are more familiar with FM than other medical practitioners [4,5]. In agreement with this view, the study participants significantly (57.7%) recommended rheumatologists to deal with FM, followed by pain management specialists (19.2%), and psychiatrists or neurologists (17.3% and 5.8%, respectively). Similarly, medical practitioners in Saudi Arabia also recommended rheumatologists, as reported by Kaki and Hazazi [14]. Another study from the United States evaluated the effect of physician specialty regarding the diagnosis and treatment of FM and reported that rheumatologists significantly exhibited higher confidence in diagnosing FM than primary care physicians [25]. However, it has been suggested that family physicians, prepared with the appropriate knowledge and experience, are best suited for managing FM [26].

Healthcare providers’ insufficient knowledge about FM may lead to high healthcare costs related to unnecessary investigations and delays in providing appropriate treatments for the patients. Most of the residents in this survey depended on undergraduate knowledge followed by postgraduate training as a learning source, while registrars and consultants learned mainly from postgraduate training and CME programs. Therefore, there is a need to improve the knowledge and training of family physicians, rheumatologists, and other medical practitioners in order to help them acquire an understanding of FM and inform them of the updated guidelines for diagnosis and treatment [10].

The study has some limitations, such as the small sample size of the study population, which has been recruited from one geographical area.

## Conclusions

The primary healthcare physicians working in the government’s primary healthcare centres in Tabuk city, Saudi Arabia, have low levels of knowledge about diagnostic criteria and treatment strategies for FM. Furthermore, the registrars followed by the residency physicians showed higher percentages of correct knowledge compared to the consultants, which may indicate defects in continuous medical education programs. Nevertheless, residents and those with a clinical experience of fewer than two years have a significantly lower level of confidence regarding diagnosing FM and differentiating it from other diseases than registrars and consultants. These findings highlight the need for continuous professional development among family physicians to improve the knowledge, attitude, and practices regarding FM in primary healthcare settings.
